# Laser Hemorrhoidoplasty: Postoperative Outcomes and Predictive Factors for Pain, Bleeding, and Recovery

**DOI:** 10.3390/life15111777

**Published:** 2025-11-19

**Authors:** Laurențiu Augustus Barbu, Nicolae-Dragoș Mărgăritescu, Liliana Cercelaru, Tiberiu Stefăniță Țenea Cojan, Mădălina Costinela Stănică, Irina Enăchescu, Ana-Maria Țenea Cojan, Valentina Căluianu, Gabriel Florin Răzvan Mogoș, Liviu Vasile

**Affiliations:** 1 Department of Surgery, Railway Clinical Hospital Craiova, University of Medicine and Pharmacy of Craiova, 2 Petru Rares Street, 200349 Craiova, Romania; laurentiu.barbu@umfcv.ro (L.A.B.); tiberiu.tenea@umfcv.ro (T.S.Ț.C.); valentina.andronache@yahoo.com (V.C.); gabriel.mogos@umfcv.ro (G.F.R.M.); 2 Department of Surgery, Emergency County Hospital, University of Medicine and Pharmacy of Craiova, 2 Petru Rares Street, 200349 Craiova, Romania; vliviu777@yahoo.com; 3 Department of Embryology and Anatomy, University of Medicine and Pharmacy of Craiova, 200349 Craiova, Romania; liliana.cercelaru@umfcv.ro; 4 Independența Vita Plus Hospital, 200738 Craiova, Romania; stanica.madalina@yahoo.com (M.C.S.); irinaenachescu@gmail.com (I.E.); 5 Faculty of Medicine, University of Medicine and Pharmacy of Craiova, 200349 Craiova, Romania; anamariatenea0324@gmail.com

**Keywords:** hemorrhoids, Goligher classification, laser hemorrhoidoplasty, diode laser, postoperative pain, bleeding, recovery, quality of life

## Abstract

**Background:** Laser hemorrhoidoplasty (LHP) is a minimally invasive alternative to excisional hemorrhoidectomy, with promising short-term outcomes. However, predictors of postoperative pain, bleeding, and recovery remain insufficiently characterized. **Methods:** We conducted a prospective study including 140 patients with Goligher grade I–IV hemorrhoidal disease (January 2020–December 2024) at Independența Vita Plus Hospital, Craiova. All patients received preoperative diosmin and topical ointments. Procedures were performed under spinal anesthesia using a standardized 1470 nm diode laser technique. Outcomes included postoperative pain (VAS), bleeding, early and late complications, recovery time, HDSS change, and quality of life assessed with a simple 0–10 numerical scale used in routine clinical practice (not a validated QoL instrument). **Results:** Mean operative time was 17.9 ± 4.8 min, with minimal blood loss. Postoperative bleeding occurred in 28.6% and was mild. Early complications increased with disease stage: 10% (II), 53% (III), 68% (IV) (*p* < 0.001). VAS pain decreased from 2.1 on day 1 to 0.3 on day 7 (*p* < 0.0001). Median recovery time was 3 days, longer in advanced stages. HDSS improved from 10.3 ± 2.5 preoperatively to 1.7 ± 1.1 at 3 months (*p* < 0.0001). QoL numerical scores also improved significantly at 3 months. Goligher grade independently predicted late bleeding (OR 70.2), high pain (OR 4.9), and prolonged recovery (OR 8.6). No recurrences were observed at 12 months. **Conclusions:** LHP provides low postoperative pain, minimal bleeding, rapid recovery, and significant symptom and QoL improvement. Disease severity strongly predicts outcomes and should guide perioperative planning.

## 1. Introduction

Hemorrhoidal disease (HD) is one of the most common benign anorectal conditions. It affects an estimated 4–40% of the general population worldwide and occurs most frequently between 45 and 65 years. In Western countries, HD generates millions of outpatient visits every year, creating a substantial healthcare and socioeconomic burden [1,2,3].

HD results from the dilatation and distal displacement of the anal vascular cushions. This mechanism leads to symptoms such as painless rectal bleeding, prolapse, pruritus ani, discomfort, and, in some cases, pain [4,5]. Conservative treatment, including dietary and lifestyle modifications or phlebotonic agents, remains the first therapeutic step. Even so, patients with persistent or advanced disease (Goligher grades II–IV) often require procedural or surgical management [1].

Traditional excisional hemorrhoidectomy, such as the Milligan–Morgan or Ferguson techniques, is effective but frequently associated with significant postoperative pain and a prolonged recovery period. Complications may include urinary retention, stenosis, bleeding, or incontinence [6,7]. To overcome these limitations, several minimally invasive alternatives have been introduced. Among them are stapled hemorrhoidopexy, Doppler-guided hemorrhoidal artery ligation, radiofrequency thermocoagulation, and laser-based techniques [8,9].

Laser-based techniques for hemorrhoidal disease include laser hemorrhoidectomy, laser hemorrhoidopexy (HeLP), and laser hemorrhoidoplasty (LHP), each offering different levels of invasiveness and clinical outcomes. Laser hemorrhoidectomy is an excisional procedure that removes hemorrhoidal tissue with laser energy; although effective, it remains associated with postoperative discomfort similar to other excisional methods [1]. Laser hemorrhoidopexy (HeLP) is a Doppler-guided, non-excisional technique that targets the terminal branches of the superior rectal artery, achieving symptom improvement with minimal pain, although its efficacy decreases in patients with significant prolapse [9]. Laser hemorrhoidoplasty (LHP), the index technique evaluated in this study, delivers laser energy into the submucosa to induce controlled coagulation and shrinkage of hemorrhoids while preserving the anoderm and mucosa. LHP has shown reduced postoperative pain, faster recovery, and favorable patient satisfaction compared with excisional or other minimally invasive therapies [5,6,7].

Laser hemorrhoidoplasty introduced in the late 2000s, is a non-excisional method in which a diode laser fiber is inserted into the hemorrhoidal tissue to induce coagulation and shrinkage of the vascular cushions. Because this approach preserves both the anoderm and the mucosa, it reduces tissue trauma and postoperative morbidity compared with conventional surgery [1,10].

Prospective studies and meta-analyses have shown that LHP offers symptom resolution and recurrence rates comparable to excisional hemorrhoidectomy. At the same time, it significantly reduces postoperative pain, bleeding, hospital stay, and time to resume daily activities [6,7,11,12].

Despite these encouraging results, the factors that predict key postoperative outcomes such as pain, bleeding, or recovery after LHP remain insufficiently clarified. Understanding these predictors may improve patient selection, perioperative planning, and shared decision-making.

The aim of this study is to prospectively assess short-term postoperative outcomes following laser hemorrhoidoplasty and to identify predictive factors for pain, bleeding, and recovery in patients with symptomatic hemorrhoidal disease. By integrating clinical outcomes with patient-reported measures, the study seeks to provide clinically relevant data that may optimize patient selection and perioperative care pathways.

## 2. Materials and Methods

### 2.1. Study Design and Ethical Approval

This prospective observational study was conducted at the Department of Surgery, Independența Vita Plus Hospital, Craiova, between 1 January 2020, and 31 December 2024. The study protocol was reviewed and approved by the Ethics Committee of Independența Vita Plus Hospital, Craiova (Approval No. 2, issued on 16 June 2025) and was carried out in accordance with the principles of the Declaration of Helsinki. Written informed consent was obtained from all participants prior to enrollment.

### 2.2. Study Population

A total of 140 consecutive patients with Goligher grade I–IV hemorrhoidal disease were enrolled. Patients with grades II–IV were included based on standard indications for surgical treatment. Patients with grade I disease were included only if they had previously received conservative therapy (oral diosmin and various topical ointments) and requested surgical intervention.

Exclusion criteria included previous anorectal surgery, coexisting anorectal pathology (anal fissure, fistula, abscess, malignancy), coagulation disorders, pregnancy, and inflammatory bowel disease. Baseline demographic and clinical data were collected, including age, sex, BMI, comorbidities, smoking status, symptom duration, and hemorrhoid grade.

### 2.3. Preoperative Assessment

All patients underwent physical examination, proctological evaluation, and anoscopy. Hemorrhoidal disease was classified according to the Goligher classification (grades I–IV). Symptom severity was assessed using the Hemorrhoidal Disease Symptom Score (HDSS; total 0–20). Disease-related quality of life (QoL) was assessed preoperatively and at 3 months using a simple 0–10 numerical scale used in routine clinical practice, where higher scores indicate worse impairment and lower scores indicate better perceived well-being. No validated standardized QoL instrument (e.g., SHS-HD) was used. Standard preoperative laboratory investigations were performed for all patients. All patients received preoperative medical therapy with oral diosmin and topical hemorrhoidal ointments to reduce hemorrhoidal congestion and improve local vascular tone.

### 2.4. Surgical Technique

All procedures were performed under spinal anesthesia to ensure patient comfort, as this approach was preferred and requested by the patients. Surgery was performed by experienced colorectal surgeons using a standardized laser hemorrhoidoplasty technique. A 1470 nm diode laser (Biolitec^®^, Jena, Germany) was used. After gentle dilation and identification of the hemorrhoidal piles, the laser fiber was introduced into the hemorrhoidal cushion through a small puncture at the mucocutaneous junction. Radial laser emission (10–12 W, 3 s pulses) was applied to induce submucosal coagulation and shrinkage of the hemorrhoidal tissue. No tissue excision was performed. Hemostasis was achieved by compression. Adjunctive mucopexy was performed selectively in patients with prolapsing hemorrhoids (grade IV).

### 2.5. Postoperative Management

All patients were discharged after 24 h of observation. Analgesia was provided with non-opioid medications as required. Standardized instructions were given regarding local hygiene, fiber supplementation, and stool softeners. The postoperative regimen included topical creams and oral diosmin. Follow-up assessments were scheduled at 1 week, 1 month, and 3 months, with additional visits in case of complications.

### 2.6. Data Collection and Outcomes

Perioperative data included operative time, estimated blood loss, and length of hospital stay. HDSS and QoL scores were recorded at baseline and at the 3-month follow-up; the primary symptom outcome was change in HDSS, while QoL change represented a secondary patient-reported outcome. Postoperative outcomes included pain intensity (VAS), early and late complications, rectal bleeding, recovery time, and QoL improvement. Early complications were defined as events occurring within 30 days after surgery (bleeding, local edema, infection, or reintervention). Late complications included delayed bleeding or recurrence after 30 days. Pain was assessed using the Visual Analogue Scale (VAS) on postoperative days 1, 3, and 7. Recovery time was defined as the number of days required to resume normal daily activities. Quality of life (QoL) was assessed preoperatively and at 3 months postoperatively using a simple 10-point numerical scale developed for routine clinical evaluation, with higher scores indicating better perceived postoperative well-being. No validated standardized QoL instrument (such as SHS-HD) was used. Postoperative outcomes including pain, complications, HDSS, and quality of life (QoL) were evaluated at 1 month and 3 months. Recurrence and late postoperative bleeding were assessed at 12 months. All postoperative assessments were recorded according to the scheduled follow-up visits.

### 2.7. Statistical Analysis

Data were analyzed using IBM SPSS Statistics v.26. Continuous variables were expressed as mean ± standard deviation (SD) or median [interquartile range (IQR)] and compared using the *t*-test, Mann–Whitney U, or ANOVA, as appropriate. Categorical variables were expressed as frequencies and percentages and compared using the χ^2^ test or Fisher’s exact test. Pain evolution over time was analyzed using the Friedman and Wilcoxon signed-rank tests. Correlations were assessed with Spearman’s rank correlation. Predictors of postoperative bleeding, pain, and prolonged recovery (>3 days) were evaluated using binary logistic regression. A *p*-value < 0.05 was considered statistically significant.

## 3. Results

A total of 140 patients were included in the study. The cohort consisted of 78 males (55.7%) and 62 females (44.3%). The mean age was 40.0 ± 11.5 years, and the mean BMI was 25.6 ± 4.1 kg/m^2^. Most patients presented with stage II hemorrhoidal disease (50%), followed by stage III (35%). The median duration of symptoms was 14 months [IQR 12–17].

There were no statistically significant differences between males and females in terms of age, BMI, hemorrhoid stage distribution, comorbidities, or symptom duration (*p* > 0.05 for all variables). Baseline demographic and clinical characteristics are presented in Table 1.

The mean operative time was 17.9 min, and the mean blood loss was 11.6 mL. All patients were discharged after one day. Early complications assessed at the 1-month follow-up occurred in 47.9% of cases, while late complications were reported in 10.7% of patients. No significant differences were observed between sexes (*p* > 0.05) (Table 2).

VAS pain scores decreased significantly from day 1 to day 7, and continued to decline until the 1-month visit. By 3 months, pain was almost completely resolved, with a mean VAS of 0.3. Early postoperative bleeding occurred within the first 30 days (28.6%), not only on day 1. Bleeding episodes consisted mainly of minimal spotting during bowel movements and resolved spontaneously within a few days. All postoperative bleeding events were mild and managed conservatively, with no need for transfusion or surgical reintervention. No recurrences were observed. Patient satisfaction also improved significantly at the 3-month follow-up (*p* < 0.001) (Table 3).

Table 4 summarizes symptom evolution from baseline to follow-up, showing marked reductions in prolapse and bleeding, rapid pain improvement, and substantial HDSS and QoL gains at the 3-month evaluation.

A clear stepwise increase was observed in operative time, postoperative pain, and early complication rates as hemorrhoid stage advanced. Patients with stage IV disease required significantly longer procedures and reported higher postoperative pain scores compared with those in stages II and III. They also experienced higher rates of early complications (*p* < 0.001). These findings underline the impact of disease severity on perioperative outcomes (Table 5).

Preoperative QoL scores increased progressively with hemorrhoid grade, showing a strong positive correlation (r = 0.879). Postoperative QoL scores improved significantly in all grades, although they remained slightly higher in grade IV. The greatest reduction in QoL scores was observed in patients with grade IV disease (Table 6).

The mean recovery time was 3.31 ± 1.20 days (median 3 [IQR 2–4]). Recovery time increased significantly with disease stage (*p* < 0.0001). Patients with complications had longer recovery times than those without (*p* < 0.0001) (Table 7).

Pain decreased significantly from day 1 to day 7 (median VAS 2 → 0; *p* < 0.0001). Goligher grade strongly correlated with operative time (r = 0.83) and recovery time (r = 0.61). Pain correlated with postoperative QoL impairment (r = 0.59). QoL improvement was similar between males and females (*p* = 0.30), and between patients with and without comorbidities (*p* = 0.91).

HDSS outcomes. The HDSS decreased markedly from baseline to 3 months in the whole cohort (N = 140): 10.27 ± 2.47 pre-op vs. 1.72 ± 1.09 post-op; median change 8.0 [IQR 7.0–10.0]; Wilcoxon *p* < 0.0001. By Goligher grade, mean HDSS improved as follows (pre→post): grade I 5.12 → 0.00 (n = 8), grade II 9.00 → 1.21 (n = 70), grade III 11.71 → 2.33 (n = 49), grade IV 14.85 → 3.23 (n = 13); all within-group Wilcoxon *p* < 0.0001.

No recurrences were recorded during the 12-month follow-up period, so Kaplan–Meier survival analysis was not applicable. Goligher grade was a significant predictor of both late postoperative bleeding (*p* = 0.006) and higher postoperative pain (VAS ≥ 3; *p* = 0.005). No significant associations were identified for sex, age, or operative time (Table 8).

Late bleeding was evaluated at the 12-month follow-up. Goligher grade was the only significant predictor of prolonged recovery (*p* = 0.001). Sex, age, and complications were not associated with delayed recovery (Table 9).

## 4. Discussion

In this prospective study involving 140 patients, laser hemorrhoidoplasty was associated with favorable short-term postoperative outcomes, including low pain scores, minimal bleeding, rapid recovery, and significant improvements in quality of life. These findings are consistent with previous studies reporting reduced postoperative morbidity and faster convalescence compared with conventional excisional hemorrhoidectomy [11,12]. Notably, Goligher grade emerged as the single most important predictor of pain, bleeding, and recovery time, highlighting its central role in perioperative risk stratification. This is one of the few studies to analyze these relationships using multivariate models, providing new evidence to guide patient selection and perioperative management in clinical practice.

### 4.1. Principal Findings

The results of this study confirm that laser hemorrhoidoplasty provides favorable postoperative outcomes in terms of pain control, bleeding, complication rates, and recovery time, reinforcing previously published evidence supporting the advantages of this minimally invasive technique over traditional excisional surgery.

The data obtained in this study further strengthen the evidence base supporting laser hemorrhoidoplasty as a safe and effective minimally invasive option for patients with symptomatic hemorrhoidal disease. The study cohort was balanced in terms of sex distribution (55.7% male, 44.3% female) with a mean age of 40 years, and most patients presented with Goligher stage II (50%) or stage III (35%) disease, reflecting a typical clinical population.

Perioperative parameters confirmed the minimally invasive nature of the procedure: the mean operative time was 17.9 min, with minimal blood loss (mean 11.6 mL) and a uniform one-day hospital stay. Early postoperative pain was low (mean VAS 2.1 on day 1), declining rapidly to near-zero values by day 7. Bleeding occurred in 28.6% of patients but was mild and self-limiting in all cases. Early and late complications were minor and manageable, and no recurrences were recorded during the 12-month follow-up period. The mean recovery time was 3.3 ± 1.2 days, underscoring the feasibility of this approach in outpatient or short-stay settings.

Importantly, hemorrhoid grade was identified as the sole independent predictor of postoperative outcomes, including pain intensity, bleeding risk, and recovery time. Patients with higher-grade disease required longer operative times and experienced more pronounced—but still clinically acceptable—symptoms during recovery.

These findings are consistent with previously published data showing lower pain, reduced bleeding, and faster recovery after LHP compared to conventional excisional hemorrhoidectomy [7,10,12,13]. In relation to stapled techniques, previous studies have emphasized the importance of strict technical accuracy to optimize outcomes. Calomino and colleagues demonstrated that careful attention to the technical details of the Longo procedure can significantly improve long-term results and reduce postoperative complications, confirming the high sensitivity of this technique to technical variations [14]. Moreover, the results confirm that LHP can be effectively and safely applied across different healthcare settings, including outpatient surgery and potentially resource-limited environments.

Overall, the present study reinforces the role of LHP as a minimally invasive and patient-friendly alternative to traditional hemorrhoidectomy, offering rapid recovery and minimal morbidity, while maintaining excellent short-term control of symptoms.

### 4.2. Comparison with Previous Studies

#### 4.2.1. Postoperative Pain

Postoperative pain is a major determinant of recovery after hemorrhoid surgery. In our cohort, pain decreased rapidly during the first postoperative week, with significant reductions between days 1, 3, and 7 (*p* < 0.0001), indicating a short-lived postoperative discomfort after LHP. Higher Goligher grades were associated with greater pain intensity (*p* < 0.001), while demographic factors had no effect. Multivariate analysis confirmed Goligher grade as the only independent predictor of clinically relevant pain (VAS ≥ 3; OR 4.90, 95 percent CI 1.60–15.02, *p* = 0.005). Pain also correlated with early postoperative QoL impairment (r = 0.586, *p* < 0.0001).

This pain pattern aligns with European and Asian studies, in which pain peaks within 24 h and decreases rapidly thereafter [1,7]. In contrast, excisional hemorrhoidectomy often causes substantial discomfort for 1–2 weeks [8]. Meta analyses consistently show lower VAS scores after LHP compared with excisional surgery, with mean differences of about 2 points on postoperative day 1 and sustained benefit through the first week [7,15]. This advantage reflects the non-excisional design of LHP, which preserves the anoderm and avoids external wounds, reducing nociceptor activation [10].

Pain in our cohort was managed with simple non-opioid analgesics, supporting the safety of outpatient LHP without intensive postoperative care. Evidence suggests that laser wavelength and energy parameters may influence postoperative discomfort, highlighting the need for standardized technique. The association between pain and early QoL impairment further underscores its impact on recovery.

These findings are consistent with reports of reduced analgesic use and earlier return to daily activities after LHP [6,13,16]. Although patients with stage IV disease had higher pain scores, discomfort remained markedly lower than typically reported after excisional hemorrhoidectomy. Overall, LHP integrates well into fast-track recovery protocols, minimizing pain-related morbidity and improving patient experience [17].

#### 4.2.2. Bleeding and Early Complications

Postoperative bleeding remains a recognized concern in hemorrhoidal surgery. Conventional excisional techniques report early or delayed bleeding rates of 10–25 percent, with some patients requiring transfusion or surgical revision [8]. By contrast, multiple studies and meta analyses have demonstrated substantially lower bleeding rates after laser hemorrhoidoplasty, typically between 0 and 7.6 percent, with events generally mild and self-limiting [1,10,18]. A large randomized trial reported early bleeding in 11.8 percent of patients at 24 h and 17.1 percent at day 7, with near-complete resolution by 6 weeks [19].

In our cohort, postoperative bleeding occurred in 28.6 percent of patients but was uniformly mild and managed conservatively. Bleeding showed a strong association with disease severity, increasing from 10.0 percent in stage II to 53.0 percent in stage III and 68.0 percent in stage IV hemorrhoids (*p* < 0.001). Multivariate analysis identified Goligher grade as the only independent predictor of late postoperative bleeding (OR 70.2, 95 percent CI 3.3–1498.8, *p* = 0.006), whereas demographic factors and operative time were not associated with bleeding risk. This likely reflects increased vascularity and mucosal fragility in advanced disease.

These findings are consistent with prior reports showing that bleeding after LHP is typically minor and self-limited [20]. Observational studies and meta analyses document low overall complication rates (<10 percent) and no major hemorrhagic events. Minor complications such as transient edema, superficial burns, or thrombosis occur infrequently (<5 percent) and are easily managed, while serious complications like stenosis or incontinence are rarely reported.

Clinically, distinguishing true post-hemorrhoidectomy bleeding from gastrointestinal bleeding due to alternative causes, including colorectal neoplasia, is essential because diagnostic and therapeutic pathways differ substantially [21,22,23]. Postoperative edema and pain should also be monitored carefully, as they may indicate secondary anorectal pathology with potential for deep infectious extension [24].

The absence of severe bleeding supports the suitability of LHP for outpatient or day-case settings. Recognizing disease severity as a risk factor may help guide perioperative management, including closer monitoring in advanced-stage patients. Overall, these results reinforce the safety and predictability of LHP in the early postoperative period.

#### 4.2.3. Recovery and Return to Normal Activity

Early return to daily activities is increasingly recognized as an important outcome after proctologic surgery. Several studies have reported that patients undergoing LHP typically resume normal activities within a few days after surgery [6,24]. This is in stark contrast to traditional hemorrhoidectomy, where convalescence may last one to three weeks [8].

In our cohort, recovery time showed a clear stage-dependent pattern: patients with stage II disease resumed normal activities earlier, while those with stage IV required approximately 4–5 days to fully recover (*p* < 0.0001). This gradient mirrors the patterns observed for postoperative pain and bleeding, emphasizing the central role of disease severity in determining recovery speed.

Patients with early postoperative complications experienced longer recovery times than those with an uncomplicated course (*p* < 0.0001), although the delay was modest, confirming that most complications after LHP are mild and self-limiting. Logistic regression analysis identified Goligher grade as the only independent predictor of prolonged recovery (>3 days; OR 8.58, 95% CI 2.45–30.12, *p* = 0.001), while demographic factors and complications did not significantly influence recovery time. A strong positive correlation between Goligher grade and recovery time (r = 0.607, *p* < 0.0001) further supports these findings.

Rapid convalescence after LHP has important clinical and socioeconomic implications. Shorter recovery facilitates early ambulation, reduces hospital stays, and allows earlier return to work. This makes LHP particularly attractive for outpatient or short-stay settings, as also demonstrated in previous reports [13].

#### 4.2.4. Comparative Evidence on Return to Normal Activity

Early return to normal activity is an important determinant of patient satisfaction and a key performance indicator in modern proctologic surgery. Multiple studies have shown that LHP provides substantially faster recovery than conventional excisional hemorrhoidectomy. A three-arm randomized controlled trial reported a median time to resumption of normal activity of 15 days after LHP compared with 30 days after excisional hemorrhoidectomy (*p* < 0.001), emphasizing the advantages of non-excisional techniques [25]. Similarly, a prospective comparative study in Annals of Coloproctology documented earlier return to work (6 vs. 13 days, *p* = 0.007) and shorter hospitalization in the LHP group, supporting its suitability for day-case surgery [6,8].

Real-world data from resource-limited settings show even faster recovery. In a prospective Bangladeshi cohort, patients resumed normal activity after a mean of 1.33 ± 0.55 days with an average hospital stay of one day [13]. Recent systematic reviews and consensus statements corroborate these findings, reporting accelerated convalescence, shorter hospitalization, and reduced analgesic use without compromising clinical outcomes [19,26].

Our results align with this evidence and confirm a stage-dependent recovery pattern. Patients with stage II disease recovered most rapidly, whereas those with stage IV required approximately 4–5 days to resume normal activities. Complications were associated with only modest delays, reflecting their generally mild and self-limiting nature. Logistic regression identified Goligher grade as the sole independent predictor of prolonged recovery (>3 days), consistent with previous studies highlighting disease severity as a major determinant of postoperative outcomes after minimally invasive hemorrhoidal procedures [10].

The rapid convalescence associated with LHP has significant clinical and socioeconomic implications. Shorter recovery supports early ambulation, reduces hospital stay, and facilitates faster return to work, making LHP well suited for outpatient and short-stay practice. Incorporating Goligher grade into preoperative counseling and perioperative planning may optimize patient selection and expectations. This recovery profile also positions LHP as a strong candidate for fast-track or ERAS pathways in proctologic surgery.

HDSS outcomes in our cohort showed a marked and clinically meaningful reduction in symptom burden at 3 months, with a median improvement of 8 points (*p* < 0.0001). These results are comparable to previously published LHP series reporting mean reductions of 6–9 points. Improvement was observed across all disease stages, with near-complete resolution in grade I and substantial benefit in grades III–IV, although patients with advanced disease retained slightly higher postoperative scores. These findings support HDSS as a sensitive measure of treatment response and confirm that LHP provides rapid and significant symptomatic relief.

#### 4.2.5. Quality of Life Outcomes

Quality of life (QoL) has become an important patient-reported outcome in the assessment of hemorrhoidal disease, complementing symptom-based scales such as the HDSS by capturing the functional and psychosocial burden of the condition on daily activities, hygiene, sleep, work, and emotional well-being [27].

Multiple studies have shown significant and durable QoL improvements after minimally invasive procedures, particularly laser hemorrhoidoplasty. Preoperative QoL scores typically range from 6 to 8 out of 10, while postoperative values decrease to 1–2 within 1–3 months, reflecting mean reductions of 5–6 points [3,11,27,28,29]. Brusciano et al. reported sustained improvements at 3 and 12 months, with comparable results also observed after HeLP and ALT procedures [11].

In our cohort, QoL improved by a mean of 5.1 points, consistent with previously published data. The largest gains were seen in patients with Goligher grade IV (mean decrease 6.0 ± 0.6), indicating that LHP remains effective even in advanced disease. QoL improvement was independent of sex and comorbidities, supporting its broad applicability. Preoperative QoL showed a strong correlation with disease severity (r = 0.879), while postoperative improvement correlated closely with pain reduction (r = 0.586, *p* < 0.0001), underscoring the link between symptom control and functional recovery.

These findings highlight the value of integrating QoL assessment alongside traditional clinical endpoints such as pain, bleeding, and complications. Routine QoL evaluation may enhance preoperative counseling, guide patient selection, and provide a more patient-centered understanding of treatment effectiveness. Future studies should further define long-term QoL trajectories and their role in individualized management strategies.

### 4.3. Clinical Implications

The clinical implications of our findings are substantial. LHP provides a minimally LHP is a minimally invasive, effective, and safe treatment option for symptomatic hemorrhoidal disease, particularly in patients with Goligher grade II–III. Its low postoperative pain, minimal bleeding, and rapid recovery make it an appealing alternative to conventional excisional hemorrhoidectomy [7,20].

A key finding of this study is the identification of Goligher grade as the only independent predictor of postoperative pain, bleeding, and prolonged recovery. This simple stratification enables personalized perioperative planning. Patients with stage II disease are well suited for day-case surgery with minimal postoperative care, whereas those with stage IV may benefit from closer monitoring, optimized analgesia, or adjunctive procedures such as LHP combined with mucopexy [9,13,30,31]. Standardized scoring tools such as HDSS and SHS can further enhance patient selection and outcome assessment [4].

The combination of short hospitalization, low analgesic requirements, and early mobilization positions LHP within enhanced recovery after surgery (ERAS) pathways, improving efficiency in both high-resource and district hospitals [6,10]. Its consistent performance in low-resource settings further supports its adaptability [9].

Incorporating patient-reported outcomes, including quality of life and treatment satisfaction, highlights the broader clinical value of LHP. These dimensions, together with its safety profile and flexibility, support its role as a first-line surgical option for appropriately selected patients [1].

Regarding advanced disease, LHP remains effective for grade III and selected grade IV hemorrhoids, although adjunctive mucopexy may be necessary in cases with significant prolapse. This was reflected in our cohort, where mucopexy was used selectively in more advanced prolapse. For patients with circumferential or bulky grade IV prolapse, stapled hemorrhoidopexy or excisional techniques may yield better functional outcomes. The choice of procedure should therefore be individualized based on prolapse severity and anatomical considerations.

Although LHP requires laser-specific equipment and disposable fibers, increasing upfront costs compared with conventional hemorrhoidectomy or rubber band ligation, its overall value may be favorable. Reduced postoperative pain, lower analgesic needs, fewer complications, and rapid return to normal activity translate into shorter hospital stays and reduced indirect costs for patients and healthcare systems. These advantages suggest potential cost-effectiveness, especially where prolonged recovery carries substantial economic burden. Nonetheless, formal cost-effectiveness analyses remain limited, and further studies are needed to better define the economic profile of LHP.

### 4.4. Strengths and Limitations

This study has several strengths. It provides prospective real-world data on the postoperative course after laser hemorrhoidoplasty in a relatively large and homogeneous cohort. All procedures were performed using a standardized technique by experienced surgeons, ensuring procedural consistency. Moreover, this is one of the few studies to analyze predictive factors for postoperative pain, bleeding, and recovery using multivariate models, which strengthens the validity of the findings. The systematic evaluation of pain, bleeding, recovery time, and quality of life offers a comprehensive perspective on short-term postoperative outcomes.

However, the study also has important limitations. First, it was conducted at a single center with a limited sample size, which may restrict the external validity and generalizability of the results. This limitation is shared by most published LHP series, which are predominantly single-center observational studies with moderate cohort sizes [11].

Second, the follow-up period was relatively short, preventing a full assessment of recurrence rates and long-term complications. A major limitation of this study is the relatively short follow-up period of 12 months, which does not allow a definitive assessment of long-term recurrence. Although no recurrences were observed within the first year, longer follow-up is necessary to accurately evaluate the durability of the procedure. This is particularly relevant because recurrence rates reported in the literature range between 0% and 11.3% [1,32]. Third, variations in laser energy settings, fiber types, and the use of adjunctive procedures (such as mucopexy) may influence outcomes and complicate direct comparisons with other published studies.

Fourth, the study did not include a control group undergoing conventional hemorrhoidectomy, which limits the ability to make direct comparative conclusions between LHP and standard excisional techniques. This is a common limitation in most published studies, with only a few randomized controlled trials available. Another limitation is the use of a non-standardized 10-point scale for the evaluation of postoperative quality of life. Although this simple scale allowed rapid clinical assessment, it does not provide the level of standardization or cross-study comparability offered by validated QoL instruments.

A further limitation is the absence of an a priori sample size calculation. Because this was a prospective observational study with consecutive enrollment during a predefined period, no primary endpoint-based estimation of the required sample size was performed. We acknowledge that defining a primary outcome and calculating the appropriate sample size would have strengthened the methodological rigor of the study.

Finally, the study was not powered to evaluate rare complications or cost-effectiveness outcomes. Economic data remain scarce in the current literature, and further studies integrating these endpoints are needed to fully establish the role of LHP in modern treatment algorithms. Future multicenter randomized controlled trials with larger sample sizes, longer follow-up periods, and direct comparison with conventional techniques are warranted to confirm these findings and better define the long-term position of LHP in the management of hemorrhoidal disease.

### 4.5. Future Directions

Future research should address several key gaps to better define the role of laser hemorrhoidoplasty in the management of hemorrhoidal disease. High-quality, adequately powered randomized controlled trials comparing LHP with conventional excisional hemorrhoidectomy are needed to evaluate postoperative pain, bleeding, recovery, recurrence, and cost-effectiveness [32,33]. These studies should include patient-reported outcomes using standardized instruments such as the SHS-HD, HDSS-QoL, SF-36, or EQ-5D to ensure consistent and comparable assessments across populations.

Predictive factors identified in this study, especially the role of Goligher grade, require validation in larger and more diverse cohorts. Developing predictive models incorporating standardized symptom scores such as HDSS and SHS may improve patient stratification and facilitate cross-study comparability [4,34].

Standardization of LHP technical parameters—including energy settings, fiber type, number of treated piles, and the adjunctive use of mucopexy—remains essential to reduce heterogeneity and optimize outcomes [13,35]. Emerging evidence that wavelength selection (980 vs. 1470 nm) may influence efficacy and recurrence further underscores the need for procedural standardization [35].

Long-term follow-up is necessary to assess recurrence, late complications, and durability of symptom control. While current evidence demonstrates favorable short-term results, robust data beyond 12–24 months are limited [35].

Cost-effectiveness analyses are also required to clarify the economic value of LHP relative to conventional surgery, particularly in resource-limited settings where reduced hospitalization and faster recovery may provide significant advantages [20,36].

Finally, multicenter and international collaborations are needed to strengthen external validity and inform evidence-based guidelines. Broader representation across healthcare systems, including low- and middle-income countries, will support the development of standardized treatment algorithms for optimal integration of LHP into clinical practice [37].

## 5. Conclusions

Laser hemorrhoidoplasty (LHP) has proven to be a safe, effective, and minimally invasive technique for the treatment of symptomatic hemorrhoidal disease. In this prospective study, LHP was associated with low postoperative pain, minimal bleeding, rapid recovery, and significant improvements in quality of life, reinforcing previously published evidence and demonstrating its applicability in both high-resource and resource-limited settings. Importantly, the procedure showed a uniformly favorable safety profile, allowing for early discharge and minimal postoperative care, supporting its use in modern day-case and fast-track surgical pathways.

Goligher grade emerged as the only independent predictor of pain, bleeding, and recovery, highlighting its central role in perioperative risk stratification and individualized management. This simple and widely used clinical staging system can guide patient selection, optimize perioperative planning, and support shared decision-making. These findings support the integration of LHP as a first-line surgical option for appropriately selected patients with hemorrhoidal disease, particularly those with grades II–III. Future multicenter randomized controlled trials with longer follow-up are warranted to validate these results, refine technical protocols, and establish standardized treatment algorithms for broader clinical implementation.

## Figures and Tables

**Table 1:** Baseline demographic and clinical characteristics of the study population (N = 140).

Variable	Total (N = 140)	Male (*n* = 78)	Female (*n* = 62)	*p*-Value
Age, years (mean ± SD)	40.0 ± 11.5	39.7 ± 11.1	40.5 ± 12.1	**0.664**
BMI (mean ± SD)	25.6 ± 4.1	25.6 ± 4.0	25.7 ± 4.3	**0.824**
Hemorrhoid stage (I/II/III/IV)	8/70/49/13	5/39/27/7	3/31/22/6	**0.982**
Comorbidities (Yes/No)	82/58	46/32	36/26	**1.000**
Symptom duration, months (median [IQR])	14 [12–17]	14 [12–16]	15 [12–18]	**0.432**

Legend: Data are presented as mean ± SD, median [IQR], or n (%). *p*-values were calculated using *t*-test, Mann–Whitney U test, or χ^2^ test, as appropriate. *p* < 0.05 was considered statistically significant. Abbreviations: BMI, body mass index; SD, standard deviation; IQR, interquartile range.

**Table 2:** Perioperative and postoperative parameters (N = 140).

Parameter	Mean ± SD/%	Min–Max	*p*-Value
Operative time (min)	17.9 ± 4.8	7–35	**0.416**
Estimated blood loss (mL)	11.6 ± 5.7	5–30	**0.367**
Hospital stay (days)	1.0 ± 0.0	1–1	**1.000**
Postoperative pain (VAS day 1)	2.1 ± 1.2	0–6	**0.941**
Early complications (%)	47.9%	—	**0.533**
Late complications (%)	10.7%	—	**1.000**

Legend: Data are presented as mean ± SD, Min–Max, or %. *p*-values were calculated using *t*-test, Mann–Whitney U test, or χ^2^ test, as appropriate. *p* < 0.05 was considered statistically significant. Note: Early complications include postoperative bleeding, local edema, infection, and reintervention within 30 days after surgery. Late complications include delayed bleeding. Min–Max indicates the minimum and maximum observed values (e.g., 7–35 min). Abbreviations: VAS, visual analogue scale; SD, standard deviation.

**Table 3:** Postoperative evolution and follow-up (N = 140).

Parameter	Preoperative	Postoperative (Early, Within 30 Days)	Postoperative (3 Months)	*p*-Value
**VAS pain** score (mean ± SD)	4.34 ± 2.53	2.06 ± 1.24	0.30 ± 0.50	**<0.001** (Wilcoxon)
Rectal bleeding (%)	0.0%	28.6%	—	**<0.001** (McNemar)
Recurrence (%)	—	—	0.0%	—
Patient satisfaction (QoL score)	6.79 ± 0.96	—	1.71 ± 0.80	**<0.001** (paired *t*-test)

Legend: Data are presented as mean ± SD or %. *p*-values were calculated using Wilcoxon signed-rank test, McNemar test, or paired *t*-test, as appropriate. *p* < 0.05 was considered statistically significant. Abbreviations: VAS, visual analogue scale; SD, standard deviation; QoL, simple 0–10 numerical quality-of-life score (0 = best, 10 = worst; nonvalidated). This table summarizes postoperative VAS pain scores, early bleeding events, and QoL changes.

**Table 4:** Evolution of symptoms from baseline to follow-up.

Parameter	Baseline	1 Month	3 Months	12 Months
Prolapse (% yes)	74%	markedly reduced	minimal	0% recurrence
Rectal bleeding (%)	90%	28.6% (early bleeding, ≤30 days)	<2%	0% late bleeding
Pain (VAS)	4.3 ± 2.5	~0.5	0.3 ± 0.5	—
HDSS (mean ± SD)	10.27 ± 2.47	—	1.72 ± 1.09	—
QoL score (0–10)	6.8 ± 1.0	—	1.7 ± 0.8	—

Early bleeding = any rectal bleeding within 30 postoperative days; late bleeding = after day 30. All bleeding episodes in this cohort were mild, self-limiting, and required only local care (no transfusions, no reinterventions).

**Table 5:** Comparative analysis between subgroups according to hemorrhoid stage (N = 140).

Variable	Stage II (*n* = 70)	Stage III (*n* = 49)	Stage IV (*n* = 13)	*p*-Value
Operative time (min)	15.44 ± 2.36	20.16 ± 2.49	27.31 ± 3.88	**<0.001** (ANOVA)
Postoperative pain (VAS day 1)	1.50 ± 0.85	2.65 ± 0.93	3.85 ± 1.21	**<0.001** (ANOVA)
Early complications (%)	10.0%(7/70)	53%(26/49)	68%(9/13)	**<0.001 (χ^2^)**

Legend: Data are presented as mean ± SD or %. *p*-values were calculated using one-way ANOVA for continuous variables and χ^2^ test for categorical variables. *p* < 0.05 was considered statistically significant. Note: Early complications include postoperative bleeding, local edema, infection, and reintervention. A clear gradient was observed with increasing hemorrhoid stage.

**Table 6:** Correlation between Goligher grade and QoL scores (N = 140).

Goligher Grade	QoL Preop (Mean ± SD)	QoL Postop (Mean ± SD)	Mean Decrease
I	5.50 ± 0.53	0.12 ± 0.35	−5.38 ± 0.52
II	6.19 ± 0.39	1.34 ± 0.48	−4.84 ± 0.50
III	7.39 ± 0.49	2.27 ± 0.49	−5.12 ± 0.70
IV	8.62 ± 0.51	2.62 ± 0.65	−6.00 ± 0.58

Legend: Data are presented as mean ± SD. Pearson correlation coefficients were calculated between Goligher grade and QoL scores. *p* < 0.05 was considered statistically significant. Abbreviations: QoL, quality of life; SD, standard deviation. This table shows pre- and postoperative QoL scores across hemorrhoid grades.

**Table 7:** Postoperative recovery time (N = 140).

Subgroup	Mean ± SD (Days)	Median [IQR]	*p*-Value
Overall	3.31 ± 1.20	3 [2–4]	**—**
Stage I	1.75 ± 0.46	2 [1.75–2.00]	
Stage II	2.83 ± 0.88	3 [2–3]	
Stage III	3.94 ± 1.18	4 [3–5]	
Stage IV	4.46 ± 0.78	4 [4–5]	**3.05 × 10^−11^ (Kruskal–Wallis)**
Without complications	2.73 ± 0.93	3 [2–3]	
With complications	3.94 ± 1.15	4 [3–5]	**1.68 × 10^−9^ (Mann–Whitney U)**

Legend: Values are presented as mean ± standard deviation and median [interquartile range]. *p*-values were calculated using Kruskal–Wallis test (stage comparison) and Mann–Whitney U test (complications). Post-hoc pairwise comparisons between stages were performed with Bonferroni correction. This table summarizes recovery time in relation to disease stage and the presence of complications.

**Table 8:** Logistic regression—Predictors of late bleeding and high postoperative pain (N = 140).

Predictor	Outcome: Late Bleeding (OR [95% CI])	*p*-Value	Outcome: VAS ≥ 3 (OR [95% CI])	*p*-Value
Goligher grade	70.2 (3.3–1498.8)	**0.006**	4.90 (1.60–15.02)	0.005
Sex (F vs. M)	1.44 (0.17–12.19)	0.735	0.99 (0.40–2.42)	0.983
Age	1.02 (0.93–1.12)	0.681	1.03 (0.99–1.07)	0.117
Operative time	1.24 (0.80–1.94)	0.339	1.14 (0.95–1.37)	0.147
VAS day 1	0.82 (0.24–2.81)	0.751	—	—

Legend: Binary logistic regression models were performed with late bleeding and high postoperative pain (VAS ≥ 3) as dependent variables. OR = odds ratio; CI = confidence interval. This table shows predictors of late bleeding and postoperative pain.

**Table 9:** Logistic regression—Predictors of prolonged recovery (>3 days, N = 140).

Predictor	Coefficient (β)	OR (expβ)	95% CI for OR	*p*-Value
Goligher grade	2.15	8.58	2.45–30.12	**0.001**
Sex (F vs. M)	0.31	1.36	0.61–3.07	0.451
Age	0.01	1.01	0.97–1.04	0.672
Complications (yes/no)	−0.45	0.64	0.15–2.78	0.546

Legend: Binary logistic regression was performed with prolonged recovery (>3 days) as the dependent variable. OR = odds ratio; CI = confidence interval. This table shows demographic and clinical differences between patients with and without pain on day 7.

## Data Availability

The data presented in this study are available on request from the corresponding author. The data are not publicly available due to patient confidentiality.
